# Applications of Artificial Intelligence for the Diagnosis of Gastrointestinal Diseases

**DOI:** 10.3390/diagnostics11091575

**Published:** 2021-08-30

**Authors:** Silvia Pecere, Sebastian Manuel Milluzzo, Gianluca Esposito, Emanuele Dilaghi, Andrea Telese, Leonardo Henry Eusebi

**Affiliations:** 1 Digestive Endoscopy Unit, Fondazione Policlinico Universitario A. Gemelli IRCCS, Università Cattolica del Sacro Cuore, 00135 Rome, Italy; sebastian.m.milluzzo@gmail.com; 2 Center for Endoscopic Research Therapeutics and Training (CERTT), Catholic University, 00168 Rome, Italy; 3 Fondazione Poliambulanza Istituto Ospedaliero, 25121 Brescia, Italy; 4 Department of Medical-Surgical Sciences and Translational Medicine, Sant’Andrea Hospital, Sapienza University of Rome, 00168 Rome, Italy; gianluca.esposito@uniroma1.it (G.E.); emanuele.dilaghi@uniroma1.it (E.D.); 5 Department of Gastroenterology, University College London Hospital (UCLH), London NW1 2AF, UK; andrea.telese@nhs.net; 6 Division of Gastroenterology and Endoscopy, IRCCS Azienda Ospedaliero-Universitaria di Bologna, 40121 Bologna, Italy; 7 Department of Medical and Surgical Sciences, University of Bologna, 40121 Bologna, Italy

**Keywords:** artificial intelligence, radiomics, deep learning, gastrointestinal lesions, gastrointestinal cancers

## Abstract

The development of convolutional neural networks has achieved impressive advances of machine learning in recent years, leading to an increasing use of artificial intelligence (AI) in the field of gastrointestinal (GI) diseases. AI networks have been trained to differentiate benign from malignant lesions, analyze endoscopic and radiological GI images, and assess histological diagnoses, obtaining excellent results and high overall diagnostic accuracy. Nevertheless, there data are lacking on side effects of AI in the gastroenterology field, and high-quality studies comparing the performance of AI networks to health care professionals are still limited. Thus, large, controlled trials in real-time clinical settings are warranted to assess the role of AI in daily clinical practice. This narrative review gives an overview of some of the most relevant potential applications of AI for gastrointestinal diseases, highlighting advantages and main limitations and providing considerations for future development.

## 1. Introduction

Continuous innovations have allowed to improve many aspects of gastroenterologists daily clinical practice, from increasing early-stage diagnoses to expanding therapeutic boundaries. In the last decades, a great deal of attention has been focused on the development computer assisted systems that could be applied in endoscopy, radiology, and pathology to improve the diagnosis, treatment, and prognosis of many gastrointestinal diseases. Indeed, machine learning has evolved in recent years due to the usage of convolutional neural networks (CNN), the improvement of the training of such networks that build the basis of artificial intelligence (AI), the development of powerful computers with advanced graphics processing, and their increasing use in many diagnostic fields. However, although AI has been applied in a wide range of gastrointestinal diseases, high-quality studies that compare the performance of AI networks to human health care professionals are lacking, especially studies with prospective design and that are conducted in real-time clinical settings.

This narrative review will give an overview of some of the most relevant potential applications of AI for both upper and lower gastrointestinal diseases (Table 1), highlighting advantages and main limitations and providing considerations for future development.

## 2. Upper Gastro-Intestinal Tract

### 2.1. Esophagus

Accumulating evidence shows the potential benefits of computer assistance (CA) in the management of esophageal conditions, such as Barrett’s esophagus (BE) and esophageal adenocarcinoma (EAC) [1]. In recent years, the ARGOS project has developed, validated, and benchmarked a computer-aided detection (CAD) system that could assist endoscopists in the detection and delineation of Barrett’s neoplasia. In their study, De Groof et al. showed that their system achieved a higher diagnostic accuracy compared to non-expert endoscopists, and it was potentially fast enough to be used in real time, taking 0.24 s to classify and delineate a Barrett’s lesion within an endoscopic image [2]. This study was conducted using a total of five independent image datasets used to train, validate, and benchmark the system. The first two datasets were used for pre-training and training respectively; the first dataset contained 494,364 endoscopic images from all intestinal segments, and the second contained 1247 high-definition (HD) white-light imaging (WLI) of confirmed early BE neoplasia and non-dysplastic BE. A third dataset containing 297 images was used for refining the training and for internal validation. Fourth and fifth datasets containing 80 images each of early BE neoplasia and non-dysplastic BE delineated by three and six experts, respectively, were used for external validation. The fifth dataset was also used to benchmark the performance of the algorithm versus 53 general endoscopists, showing an accuracy of 88% vs. 73%, a sensitivity of 93% vs. 72%, and a specificity of 83% vs. 74%, respectively [2]. Similarly, in 2020, Hashimoto and colleagues published a single-center retrospective study on a system developed for the detection of early esophageal neoplasia in BE. The algorithm was programmed to distinguish images of lesions with or without dysplasia. A total of 916 images of early esophageal neoplasia in BE and 919 images of BE without high-grade dysplasia were used for training the system. It was validated using 458 images, 225 with dysplasia and 233 without dysplasia, reporting a sensitivity, specificity, and accuracy per image of 96.4%, 94.2%, and 95.4%, respectively. The authors also found that the specificity for images taken with advanced imaging techniques, such as narrow-band imaging (NBI) and near focus, was significantly higher than white-light imaging (WLI) and standard focus [3]. 

Dedicated models analyzing WLI and NBI images have shown high disease-specific diagnostic accuracy not only for BE and EAC [4] but also for esophageal squamous cell carcinoma (ESCC) [5]. Dedicated algorithms have also been implemented to analyze enhanced endoscopy imaging, allowing to evaluate disease-specific mucosal and vascular patterns [6], the presence of submucosal invasion [7], the depth of invasion [8], and microendoscopy use for both ESCC [9] and BE [10]. Moreover, a recent meta-analysis has shown an overall high accuracy in the detection of early EAC, with a significantly better performance compared to endoscopists in terms of the pooled sensitivity (0.94 vs. 0.82, *p* = 0.01). However, these results were based mainly on studies where endoscopic images were reviewed retrospectively, whereas data from prospective trials are more limited [11]. 

With increased and rapid development of science and technology, AI systems are becoming a method of examining and diagnosing digestive tract diseases. Preliminary data have shown possible grounds for improvement of the sensitivity of the endoscopists for detection of superficial ESCC [12] and for evaluation of the depth of mucosal invasion [13]. Guo and colleagues developed a CAD system for real-time, computer-assisted diagnosis of precancerous lesion and early ESCC. The deep-learning model demonstrated a 98% sensitivity and a 95% specificity for endoscopic images and up to 100% per-lesion sensitivity for video datasets [14]. 

A recent study was conducted on protruding lesions of the esophagus, integrating standard WL endoscopic images with endoscopic ultrasound (EUS) images [15]. The diagnostic accuracy in differentiating sub-types of protruding lesions of the AI system outperformed most of the endoscopists enrolled to interpret the images. In addition, when CA models and endoscopists predictions were combined, a higher diagnostic accuracy was achieved compared with the endoscopists alone [15]. CA has been used for image recognition of histology and pathology specimens to categorize dysplastic and non-dysplastic BE and EAC [16] and also for cytology samples obtained by wide-area transepithelial sampling (WATS3D) [17] or by Cytosponge [18], achieving promising results and matching the diagnostic performance of experienced pathologists. 

Similarly, CA has been applied to radiological techniques. In a recent study by Takeuchi and colleagues, the AI-based diagnostic system succeeded at analyzing CT images in detecting esophageal cancer with an 85% diagnostic accuracy [19]. Furthermore, AI has shown to be effective in the automated segmentation of the esophagus for radiotherapy [20] and for predicting histopathological features of aggressiveness of esophageal lesions by evaluating metabolic activity assessed via PET-CT [21]. 

Modelling of esophageal pathology would likely represent another application to resolve more complex multifactorial clinical questions, such as the presence of nodal metastasis in early EAC treated with endoscopic resection [22] or in early-stage ESCC treated with esophagectomy, identifying new indicators not included in current staging criteria [23]. 

Finally, preliminary data for other potential applications of AI, including dosimetry evaluation for radiotherapy [24], prediction of response to neoadjuvant chemoradiotherapy of ESCC [25], and early recurrence of disease after surgery [26], have provided encouraging results in the management of esophageal diseases.

### 2.2. Stomach

#### 2.2.1. AI and Helicobacter Pylori Infection

The literature search resulted in many studies specifically focused on the application of AI and *H. pylori* diagnosis. In the metanalysis of Bang et al. [27], six studies [28,29,30,31,32,33] established the AI algorithm based on CNN, whereas two studies [34,35] established support vector machine-based algorithms. In the first case, the sensitivity, specificity, and area under the curve (AUC) with 95% confidence interval (CI) of AI for the prediction of *H. pylori* infection were 87% (95% CI 72–94), 86% (95% CI 77–92), and 0.92 (95% CI 0.90–0.94), respectively, while for studies of image-based analysis [28,30,32,35], sensitivity, specificity, and AUC with 95% CI of AI for the prediction of *H. pylori* infection were 81% (95% CI 68–90), 93% (95% CI 82–98), and 0.93 (95% CI 0.90–0.95), respectively. AI showed a good performance applied to the endoscopic diagnosis of *H. pylori* infection, but its performance can only be valid for the population under evaluation (single Asian center) and depends on the prevalence of target conditions for the selected population (spectrum bias or class imbalance). In a more recent meta-analysis by Mohan et al., the study selection was restricted only to those that used a CNN-based AI algorithm [36]. The pooled accuracy, sensitivity, and specificity of AI in the diagnosis of *H. pylori* infection were 87.1% (95% CI 81.8–91.1), 86.3% (95% CI 80.4–90.6), and 87.1% (95% CI 80.5–91.7), respectively. AI appeared to perform better than the endoscopists in terms of overall accuracy; however, the lack of uniformity of the algorithm’s training process, the retrospective nature of the studies, and the lack of studies from geographical areas other than Asia represent major limits for the analysis. 

#### 2.2.2. AI and Gastric Precancerous Lesions and Gastric Cancer

The application of AI for the diagnosis of gastric precancerous conditions regards the diagnosis of chronic atrophic gastritis (CAG) [37,38] and gastric intestinal metaplasia (GIM) [39]. To improve the diagnostic rate of CAG, two main studies were conducted. In the first study by Guimaraes et al., a deep-learning (DL) system was developed with non-standardized, real-world WLI images. A first data set with 100 atrophic images of 37 patients and 100 non-atrophic images of 64 patients and a second data set of 30 atrophic images and 40 non-atrophic images were considered. The second data set was also evaluated by three expert endoscopists and three non-expert endoscopists. No statistically significant difference between the expert and the non-expert group was found. DL showed a significantly higher overall accuracy compared to the endoscopists (93% vs. 80%, *p* = 0.003) and an area under the curve of 0.98 [37]. The second study evaluated a CNN collecting a total of 5470 images of the antrum, of which 3042 presented CAG (1458 images of mild atrophic gastritis, 1348 images of moderate atrophic gastritis, and 38 images of severe atrophic gastritis). The diagnostic accuracy, sensitivity, and specificity of the CNN-CAG model for CAG were 0.9424, 0.9458, and 0.9401, respectively. The detection rates of mild, moderate, and severe atrophic gastritis were 94%, 95%, and 99%, respectively [38]. Concerning GIM diagnosis, Yan et al. [39] developed an intelligent diagnostic system using a CNN including 158 patients with a diagnosis of GIM, 622 images with magnified narrow-band imaging (M-NBI), and 426 images with NBI. On the other hand, 462 M-NBI images and 370 NBI images from 178 patients without diagnosis of GIM were also collected. Different CNN models were built, and the most efficient system showed an average accuracy, sensitivity, and specificity of 89.0%, 93.3%, and 85.4%, respectively, and was selected for the intelligent diagnostic (ID) system. To test the ID system, a per-image and a per-patient analysis were performed. Per-image analysis for NBI included 242 images of GIM lesions from 37 patients and 235 non-GIM images from 43 patients. For NBI images, the ID system showed a sensitivity of 91.2% (95% CI 83.9–95.4), a specificity of 71.1% (95% CI 59.9–80.3), and an accuracy of 82.7% (95% CI 76.6–87.7). For M-NBI images analysis, 129 GIM images and 152 non-GIM images were used. The ID system showed a sensitivity of 93.8% (95% CI 87.8–97.1), a specificity of 84.2% (95% CI 77.2–89.4), and an accuracy of 88.6% (95% CI 84.3–92.1). The area under the curve for NBI images was 0.907, whilst for M-NBI images, it was 0.941. At a per-patient level, the ID system was compared to four expert endoscopists. A total of 80 patients, 37 patients with GIM and 43 without GIM, were enrolled, and one image per patient was used. The expert endoscopists showed a sensitivity, specificity, and accuracy of 86.5% (95% CI 70.4–94.9), 81.4% (95% CI 66.1–91.1), and 83.8% (95% CI 73.8–91.1), respectively. The ID system showed a sensitivity of 91.9% (95% CI 77.0–97.9), a specificity of 86.0% (95% CI 71.4–94.2), and accuracy of 88.8% (95% CI 79.7–94.7). No statistical significance was reached even if the ID system presented slightly better results.

In the setting of AI and detection of gastric cancer (GC), in the first meta-analysis published by Lui et al. [40], diagnostic performance of AI resulted in a pooled sensitivity and specificity of 92.1% (95% CI 87.7–95.4) and 88.0% (95% CI 78.0–95), with an AUC of 0.96% (95% CI 0.94–0.99). Two more recent meta-analyses by Mohan et al. [41] and Arribas et al. [42] also provided the diagnostic performance of AI for gastric cancer detection. In the first study, the pooled accuracy of AI was 85.8% (95% CI 79.8–90.3), and the pooled sensitivity, specificity, positive predictive value (PPV), and negative predictive value (NPV) were 75.1% (95% CI 57.9–86.9), 91.4% (95% CI 84.3–95.4), 51% (95% CI 30.9–70.8), and 92.1% (95% CI 85.9–95.7), respectively. The second meta-analysis showed similar results, as AI reached a sensitivity, specificity, PPV, and NPV of 88% (95% CI 78–94), 89% (95% CI 82–93), 88% (95% CI 80–93), and 89% (95% CI 80–94), respectively. However, the majority of included studies were graded as low quality, mainly due to the high risk of selection bias in the training or validation sets and for the lack of clinical real-time validation. Therefore, Tang et al. [43] developed and validated a real-time DL-CNN system for detecting EGC that confirmed high accuracy rates of AI with sensitivity, specificity, and the AUC ranging from 85.9% to 95.5%, 81.7–90.3%, and 0.887–0.940, respectively. Finally, Wu et al. [44] carried out a multicenter, randomized, controlled trial using a deep-learning CNN and deep reinforcement-learning system named “ENDOANGEL”. Patients included in the ENDOANGEL group had significantly fewer blind spots compared with the control group (mean 5.38 (standard deviation (SD) 4.32) vs. 9.82 (SD 4.98); *p* < 0.001) and longer inspection time (5.40 (SD 3.82) vs. 4.38 (SD 3.91) minutes; *p* < 0.001). ENDOANGEL showed a per-lesion accuracy of 84.7%, sensitivity of 100%, and specificity of 84.3% for detecting gastric cancer. Concerning the use of radiomics in gastric diseases, published articles mainly focus on the use of radiomics and gastric cancer. Regarding the prediction of prognosis of gastric cancer in a preoperative setting, the study of Shin J et al. [1], published in 2021, aimed to develop a radiomics-based prognostic model for recurrence-free survival (RFS) using preoperative contrast-enhanced CT in local advanced gastric cancer. This retrospective study included a training and an external validation cohort of 349 and 61 patients who underwent curative resection for gastric cancer without neoadjuvant therapies. 

The integrated area under the curve (iAUC) values for RFS prediction were 0.616 (95% CI 0.570–0.663), 0.714 (95% CI 0.667–0.759), and 0.719 (95% CI 0.674–0.764) in clinical, radiomic, and the clinical and radiomic merged models, respectively. In external validation, the iAUC were 0.584 (95% CI 0.554–0.636), 0.652 (95% CI 0.628–0.674), and 0.651 (95% CI 0.630–0.673) in clinical, radiomic, and merged models, respectively. The radiomic model showed significantly higher iAUC values than the clinical model.

Another worthwhile issue regarding the best management to offer to the patient with a diagnosis of gastric cancer would be to define the real necessity of eventual neoadjuvant chemotherapy (NAC). A study by Wang Y et al. [45], published in 2020, aimed to investigate the role of CT radiomics for differentiation between T2- and T3/4-stage cases in gastric cancer to avoid the adverse events of NAC in those patients who should directly undergo surgery. A total of 244 consecutive patients with pathologically proven gastric cancer were retrospectively included, and a training cohort of 171 patients and a validation cohort of 73 patients were provided. Preoperative arterial and portal phase contrast-enhanced CT images were retrieved. Arterial and portal phase-based radiomics model showed areas under the curve of 0.899 (95% CI 0.812–0.955) and 0.843 (95% CI 0.746–0.914) in the training cohort and 0.825 (95% CI 0.718–0.904) and 0.818 (95% CI 0.711–0.899) in the validation cohort, respectively. The results exhibited that the radiomics models based on the CT images may provide potential value for differentiation of the depth of tumor invasion in gastric cancer. Concerning the use of radiomics, the study of Shin J et al. [46] aimed to develop a radiomics-based prognostic model for recurrence-free survival (RFS) using preoperative contrast-enhanced CT in local advanced gastric cancer. This retrospective study included a training and an external validation cohort of 349 and 61 patients who underwent curative resection for gastric cancer without neoadjuvant therapies. The integrated area under the curve (iAUC) values for RFS prediction were 0.616 (95% CI 0.570–0.663), 0.714 (95% CI 0.667–0.759), and 0.719 (95% CI 0.674–0.764) in clinical, radiomic, and merged models, respectively. In external validation, the iAUC were 0.584 (95% CI 0.554–0.636), 0.652 (95% CI 0.628–0.674), and 0.651 (95% CI 0.630–0.673) in clinical, radiomic, and merged models, respectively. The radiomic model showed significantly higher iAUC values than the clinical model. 

## 3. Lower Gastro-Intestinal Tract

### 3.1. Inflammatory Bowel Disease

Endoscopic visualization is highly reliable for the evaluation of the colonic mucosa with inflammatory bowel disease (IBD); however, histological examination is still the gold standard for diagnosis. Therefore, initially, AI had been tested to automatically evaluate the inflammation both of ulcerative colitis (UC) and Crohn’s disease (CD) in order to avoid frequent repetition of biopsies during endoscopy. 

Regarding UC, Maeda et al. [47] applied a novel AI system on 100 patients, obtaining a final diagnosis in all cases, with a reaction time of 40 ms/frame and a sensitivity, specificity, and accuracy of 74% (95% CI, 65–81), 97% (95% CI, 95–99), and 91% (95% CI, 83–95), respectively. 

Similar results were obtained by Ozawa et al. [48], who tested a novel, CNN-based AI system that had been previously trained on 26,304 colonoscopy images from a cumulative total of 841 patients with UC and then tested on 3981 images from 114 patients. The AI system was able to correctly classify 73% of Mayo 0, 70% of the Mayo 1, and 63% of the Mayo 2–3 images, with a reaction time of 20 ms/frame. The system also achieved promising results in terms of AUC when differentiating Mayo 0 from 1–3 and Mayo 0–1 versus 2–3, being 0.86 (95% CI, 0.84–87) and 0.98 (95% CI, 0.97–98), respectively. In another study by Stidham et al. [49], AI was designed to perform a binary categorization into remission (Mayo 0 or 1) and moderate/severe disease (Mayo 2 or 3); its performance was comparable to expert endoscopists, being able to distinguish endoscopic remission from moderate-to-severe disease with an AUC of 0.96 (95% CI, 0.96–0.97), PPV of 0.87 (95% CI, 0.85–0.88), sensitivity of 83.0% (95% CI, 80.8–85.4), specificity of 96.0% (95% CI, 95.1–97.1), and NPV of 0.94 (95% CI, 0.93–0.95). Histological remission was evaluated with AI system specifically designed by Takenaka et al. [50]. It was firstly trained on 40,758 images of UC colonoscopies with biopsy and then tested on 875 patients with UC. The model achieved accuracy of 90.1% (95% CI, 89.2–90.9%) and 92.9% (95% CI, 92.1–93.7%) to predict endoscopic and histological remission, respectively, without the need for mucosal biopsies. 

In the setting of CD, Klang et al. [51] trained a CNN system for automatic detection of small bowel Crohn’s ulcers that was then tested on 17,640 small bowel capsule endoscopy (SBCE) images from 49 patients. The AI system required a surprisingly short time for the analysis of the whole SBCE video (about 3.5 min median) and achieved accuracy, sensitivity, and specificity of 96.7%, 98%, and 97%, respectively. 

In addition to detecting the lesions, AI can also assist the endoscopist in the evaluation of endoscopic disease severity. This potential application was tested by Barash et al. [52] in a recent retrospective study in which the authors highlighted the low inter-observer agreement among expert endoscopists for classification of inflammatory signs, therefore underlining the importance of an automatic AI-assisted scoring system. AI achieved an overall agreement for consensus reading of 67% and 91% for distinction of mild and severe inflammation (ulcers classified as grade 1 and 3). Although these data need validation in real-life settings, and only small retrospective studies have been performed so far, results seem promising, and AI could significantly improve SBCE reading soon.

### 3.2. Polyp Detection and Colorectal Cancer

Colonoscopy is the gold standard for the identification and removal of precancerous colonic lesions, which may evolve into colorectal cancer (CRC). Among the factors affecting the effectiveness of colonoscopy, quality of bowel cleansing and adenoma detection rate (ADR) are the most relevant [53]. The role of technological improvements, and particularly AI, have been investigated in these fields. 

Zhou et al. [54] developed a novel AI system (ENDOANGEL) designed to provide precise and objective bowel preparation scores according to Boston Bowel Preparation Scale [55]. ENDOANGEL was retrospectively trained with 5476 colonoscopy images evaluated by five experts and subsequently validated on 592 untrained images. Then, 120 images from each bowel preparation classification were randomly chosen as a test dataset to compare accuracy of ENDOANGEL to human evaluation. AI showed an accuracy of 93.33%, significantly higher accuracy than all endoscopists. Furthermore, using 20 colonoscopy videos, ENDOANGEL was tested to evaluate every 30-s real-time bowel preparation with an accuracy of 89.04%.

Another CNN-based automatic quality control system (AQCS) for evaluation of withdrawal time and stability, bowel cleansing and identification of polyps was designed by Sun and co-workers [56]. The authors tested this AQCS system in a prospective RCT comparing routine colonoscopies with or without the assistance of AI. A total of 308 and 315 patients, respectively, were enrolled in the study. AQCS significantly increased all performances evaluated. In detail, AI-assisted colonoscopy group showed better ADR (0.289 vs. 0.165, *p* < 0.001), mean number of adenomas (0.367 vs. 0.178, *p* < 0.001), polyp detection rate (0.383 vs. 0.254, *p* = 0.001) and polyps detected per procedure (0.575 vs. 0.305, *p* < 0.001), withdrawal time (7.03 min vs. 5.68 min, *p* < 0.001), and adequate bowel cleansing (87.34% vs. 80.63%, *p* = 0.023).

Since a high inter-operator variability of ADR has been reported [54,55] with relevant consequences in terms of interval CRC [56], the role of AI in the improvement of diagnostic performances of colonoscopy have been largely investigated. AI was initially tested for polyp detection (CADe), serving as a virtual assistant. Preliminary studies using rudimental, hand-crafted AI algorithm obtained poor results, with low diagnostic performances [57] and high false-positives rates mainly due to artifacts [58]. Following the introduction of CNN systems, AI performances showed significant improvements. The results obtained were summarized in four recent systematic reviews and meta-analyses, which mainly agree on the relevant role of AI in the detection of polyps. In details, Aziz et al. [59] included three RCT with 2815 patients (1415 in standard colonoscopy (SC) and 1400 in AI groups) and showed that AI-assisted colonoscopy resulted in a significant improvement of ADR (32.9% vs. 20.8%, RR: 1.58, 95% CI 1.39–1.80, *p* = < 0.001) and polyp-detection rate (PDR) (43.0% vs. 27.8%, RR: 1.55, 95% CI 1.39–1.72, *p* = < 0.001) compared with SC. These data were confirmed also by Zhang and colleagues [60], who showed that AI significantly improved PDR (*p* < 0.001, OR = 1.95, 95% CI: 1.75 to 2.19, I2 = 0%) and also ADR (*p* < 0.001, OR = 1.72, 95% CI: 1.52 to 1.95, I2 = 33%), and Li et al. [61], who demonstrated that AI-assisted colonoscopy reached better performances compared to SC in terms of both PDR (OR = 1.91; 95% CI 1.68–2.16) and ADR (OR = 1.75; 95% CI 1.52–2.01). Similarly, Hassan et al. [62] highlighted that AI significantly improved the ADR (36.6% vs. 25.2%; RR, 1.44; 95% CI, 1.27–1.62; *p* < 0.01) but also the number of adenomas per colonoscopy (APC), which was higher in the CADe group compared with control (1249/2163 vs. 779/2191; RR, 1.70; 95% CI, 1.53–1.89; *p* < 0.01; I2 = 33%).

In addition to lesions detection, AI has been also investigated for automatic polyp characterization (CADx) and whether it can potentially distinguish precancerous from benign lesions, avoiding useless polyps’ removal for histological evaluation. In this setting, a pioneering study was performed by Tischendorf et al. [63] with a CADx system able to discriminate non-adenomatous from adenomatous polyps based on vascularization features with NBI magnification vision. Although good performances were obtained, human observers performed better than AI both in terms of sensitivity (93.8% vs. 90%) and specificity (85.7% vs. 70%).

Similar to CADe, CADx achieved better results with the introduction of deep-learning systems. A benchmark study in this setting was performed by Birne et al. [64], who tested an AI system on 125 polyps that were histologically defined as adenomas or hyperplastic. The AI performed a real-time evaluation of the polyps on NBI non-magnified vision according to the Narrow-band Imaging International Colorectal Endoscopic (NICE) classification [65]. The AI model did not reach enough confidence to predict the histology of 19 polyps, whereas for the remaining 106 polyps, it showed an accuracy of 94% (95% CI 86–97%), sensitivity for identification of adenomas of 98% (95% CI 92–100), specificity of 83% (95% CI 67–93), NPV of 97%, and PPV of 90%.

In a prospective study by van der Zander et al. [66], CADx diagnostic performances using high-definition white-light (HDWL) and blue-light imaging (BLI) were compared to those of optical diagnosis by expert and novice endoscopists using the BLI Adenoma Serrated International Classification (BASIC) [67]. The accuracy of AI was 88.3% using HDWL images, 86.7% using BLI images, and 95.0% using both, performing better than experts (81.7%, *p* = 0.03) and novices (66.7%, *p* < 0.001). Sensitivity was also higher for CADx (95.6% vs. 61.1% and 55.4%), whereas specificity was higher for experts compared with CADx and novices (95.6% vs. 93.3% and 93.2%). 

CADx was also evaluated using endocytoscopy (EC-CAD). This technique permits cellular nuclei visualization in vivo with ultra-magnification (×450). Mori et al. [68] reported the results of EC-CAD in four patients using EndoBRAIN (Cybernet Systems Corp., Tokyo, Japan), an AI-based system that analyzes cell nuclei, crypt structure, and micro-vessels in endoscopic images to identify colon cancers. This AI system was further investigated including a comparison between AI and humans (20 trainees and 10 expert endoscopists) [69]. Using methylene blue staining or NBI, EndoBRAIN identified colonic lesions significantly better than non-expert endoscopists, while only sensitivity and NPV were significantly higher compared to experts. Two main studies analyzed the potential application of AI in CADx for diminutive polyps [70,71,72], with promising results that were also confirmed by a recent meta-analysis [73], showing a sensitivity and specificity of 93.5% (95% CI, 90.7–95.6) and 90.8% (95% CI, 86.3–95.9), respectively. 

These good performances could justify a “resect and discard” or “diagnose and leave” strategy. In the first case, polyps are still removed but not sent for histological analysis. According to Hassan and co-workers [74], this strategy could result in an annual saving of $25/person and a total of $33 million in the United States of America, with no relevant impact on the efficacy of CRC screening. On the other hand, a “diagnose and leave” strategy could avoid the risk of unnecessary of polypectomy and spare the cost of endoscopic polypectomy, which have been approximately estimated as $179 per person, giving a total saving of $1 billion per year to the United States of America health care system [75]. However, this strategy could expose patients to the risk interval of CRC due to the misdiagnosis of precancerous colonic lesions that would be left in place. Few data are available on CAD system applied to computed tomography colonography (CTC) for detection of colorectal polyps, mainly due to the high number of false positives (FPs). To overcome the issue, Ren et al. [76] proposed a CAD-CTC scheme using shape index, multiscale enhancement filters, and a total of 440 radiomic features. This scheme was evaluated on 152 oral contrast-enhanced CT datasets from 76 patients with 103 polyps ≥ 5 mm. The detection results were encouraging, achieving a high sensitivity and maintaining a low FP rate for polyps ≥ 5 mm. In addition, a recent proof-of-concept study [77] evaluated a non-invasive, radiomics-based, machine-learning differentiation of benign and premalignant colorectal polyps in a CT colonography datasets in an asymptomatic, average-risk colorectal cancer screening cohort including 59 patients. Results showed a sensitivity of 82% (95% CI: 74–91), a specificity of 85% (95% CI: 72–95), and AUC of 0.91 (95% CI: 0.85–0.96), providing a potential basis for future prospective studies in the setting of non-invasive analysis of CT colonography-detected polyps.

## 4. Discussion and Conclusions

AI is rapidly integrating into clinical practice [78], becoming, in few years, a reliable tool for supporting physicians in the study of GI tract. This review focused on AI and diagnostic aspects (endoscopy, radiology, and pathology) of GI diseases and showed that AI seems to have a great potential in the field of detection of inflammatory, pre-cancerous, and cancerous lesions of GI tract (Table 2). 

From available data, AI seems to have high overall accuracy for the diagnosis of any neoplastic lesion, while for inflammatory disease, fewer studies have been performed but with encouraging results. Nevertheless, major limits should be carefully taken into account. First, AI performance results were sometime heterogeneous from one study to another, making it difficult to compare them [79]. Second, the size of training and test datasets varied widely across studies. Third, most CAD or CNN systems were developed in single centers, and many data come from pre-clinical studies, raising the concern of selection and spectrum bias. Finally, most of AI systems for endoscopy derived from retrospective, non-randomized setting, and standardization still remains an issue. In conclusion, AI is definitely changing our work with possible enormous potential benefits, but thresholds for guidelines for standard patient care are needed also to overcome major limitations that, to date, represent important ethical issues and obstacles for its widespread use and implementation.

## Figures and Tables

**Table 1:** Key points of AI application in GI disease.

Field	Key Points
ESOPHAGUS	1. AI seems a useful tool for detection of BE, EAC, and ESCC, although current evidence is limited by study type.
	2. Further studies could better address the role of AI for prediction of prognosis and treatment response in esophageal cancers.
STOMACH	1. CNN-based algorithms showed good diagnostic performances for HP detection.
	2. AI could improve not only lesion detection of GC but also patients’ selection for chemotherapy and definition of prognosis.
LOWER GI TRACT	1. In the field of UC and CD, AI can be used for automatic detection and grading of disease activity.
	2. CADx and CADe are currently the most promising and effective clinical application of AI.

Legend: BE, Barrett’s esophagus; EA, esophageal adenocarcinoma; ESCC, esophageal squamous cell carcinoma; HP, Helicobacter pylori; GC, gastric cancer; UC, ulcerative colitis; CD, Crohn’s disease; CADx, automatic polyp characterization; CADe, automatic polyp detection; GI, gastro-intestinal.

**Table 2:** Summary of topics investigated.

Field	Disease	Topic Investigated
ESOPHAGUS	*BE*	Detection of dysplasia
	*EAC*	Detection of lesions Depth of invasion
	*ESCC*	Detection of lesions Depth of invasion Prediction of lymph nodal invasion Guide for radiotherapy treatment Prediction of treatment response Prediction of risk of recurrence
STOMACH	*HP*	Prediction of infection
	*CAG*	Detection
	*GIM*	Detection
	*GC*	Detection Dept of invasion Prediction of recurrence
LOWER GI TRACT	*UC*	Diagnosis Disease activity evaluation
	*CD*	Ulcer detection Ulcer severity grading
	*PCL*	Detection Characterization

Legend: BE, Barrett’s esophagus; EAC, esophageal adenocarcinoma; ESCC, esophageal squamous cell carcinoma; HP, Helicobacter pylori; CAG, chronic atrophic gastritis; GIM, gastric intestinal metaplasia; GC, gastric cancer; UC, ulcerative colitis; CD, Crohn’s disease; PCL, precancerous colonic lesions; GI, gastro-intestinal.
